# NAA40 contributes to colorectal cancer growth by controlling PRMT5 expression

**DOI:** 10.1038/s41419-019-1487-3

**Published:** 2019-03-11

**Authors:** Christina Demetriadou, Demetria Pavlou, Fotios Mpekris, Charis Achilleos, Triantafyllos Stylianopoulos, Apostolos Zaravinos, Panagiotis Papageorgis, Antonis Kirmizis

**Affiliations:** 1 Epigenetics Laboratory, Department of Biological Sciences, University of Cyprus, 2109 Nicosia, Cyprus; 2 Cancer Biophysics Laboratory, Department of Mechanical and Manufacturing Engineering, University of Cyprus, 1678 Nicosia, Cyprus; 3 Tumor Viruses and Cancer Laboratory, Department of Biological Sciences, University of Cyprus, 2109 Nicosia, Cyprus; 4 Department of Life Sciences, European University Cyprus, 1516 Nicosia, Cyprus

## Abstract

N-alpha-acetyltransferase 40 (NAA40) catalyzes the transfer of an acetyl moiety to the alpha-amino group of serine 1 (S1) on histones H4 and H2A. Our previous studies linked NAA40 and its corresponding N-terminal acetylation of histone H4 (N-acH4) to colorectal cancer (CRC). However, the role of NAA40 in CRC development was not investigated. Here, we show that NAA40 protein and mRNA levels are commonly increased in CRC primary tissues compared to non-malignant specimens. Importantly, depletion of NAA40 inhibits cell proliferation and survival of CRC cell lines and increases their sensitivity to 5-Fluorouracil (5-FU) treatment. Moreover, the absence of NAA40 significantly delays the growth of human CRC xenograft tumors. Intriguingly, we found that NAA40 knockdown and loss of N-acH4 reduce the levels of symmetric dimethylation of histone H4 (H4R3me2s) through transcriptional downregulation of protein arginine methyltransferase 5 (*PRMT5*). NAA40 depletion and subsequent repression of PRMT5 results in altered expression of key oncogenes and tumor suppressor genes leading to inhibition of CRC cell growth. Consistent with this, *NAA40* mRNA levels correlate with those of *PRMT5* in CRC patient tissues. Taken together, our results establish the oncogenic function of the epigenetic enzyme NAA40 in colon cancer and support its potential as a therapeutic target.

## Introduction

In every eukaryotic cell, ~147 base pairs of DNA is wound around four core histone proteins (H3, H4, H2A, and H2B) constructing a nucleosome, which makes up the basic structural unit of chromatin. A wide spectrum of chromatin-modifying enzymes, commonly refer to as ‘writers’, decorate the globular domain and N-terminal tails of nucleosomal histones with numerous post-translational modifications (PTMs)^1^. These PTMs dictate chromatin architecture and therefore tightly regulate DNA-based processes, such as gene expression^2,3^. Histone acetyltransferases (HATs) constitute one of the most extensively studied group of epigenetic writers, which modify chromatin via the deposition of acetyl-groups on histone proteins. Importantly, deregulation of HAT enzymes significantly alters normal gene expression and is implicated in the development of several diseases including cancer^4^.

Although an extensive body of work has been accumulated over the past decades describing the role of many HATs in gene regulation and tumorigenesis, the function of some of these enzymes still remains poorly characterized^5^. One notable example is the N-alpha-acetyltransferase 40 (NAA40) enzyme that belongs to the N-terminal acetyltransferase (NAT) family of enzymes sharing the conserved sequence motif of the GCN5-related acetyltransferase superfamily^6^. Unlike all other HATs that acetylate the side chains of internal lysine residues, NAA40 (also known as NatD, Nat4, or Patt1) catalyzes the addition of an acetyl moiety to the alpha-amino group of the first amino acid residue on histones H4 (N-acH4) and H2A (N-acH2A)^7^. For years, this enzyme remained unexplored because it was thought to catalyze a non-regulatory modification. Intriguingly, studies in yeast demonstrated that NAA40 and its catalyzed N-acH4 regulate the expression of specific sets of genes controlling cell growth^8,9^. In support of this identified cellular function, several other studies have implicated NAA40 deregulation in the development and progression of different types of malignancy. In particular, a recent study has indicated that NAA40 is a critical regulator of cell invasion during lung cancer metastasis^10^. Moreover, NAA40 was shown to be downregulated in hepatocellular carcinoma tissues and ectopic NAA40 expression sensitizes hepatoma cancer cell lines to drug-induced apoptosis^11^. Conversely, we have previously unveiled a pro-survival role for NAA40 in colorectal cancer (CRC) cells suggesting that it may stimulate cancer cell growth^12^. Despite the above evidence, the contribution of NAA40 in colorectal carcinogenesis remained unclear.

Histone-modifying enzymes often cross-regulate each other in order to generate a highly dynamic interplay amongst histone modifications, which is important in defining gene expression patterns^13,14^. Consistent with this notion, we have previously reported that NAA40 and its mediated N-acH4 inhibit the activity of the histone arginine methyltransferase HMT1 toward arginine 3 of histone H4 (H4R3) to control ribosomal gene expression in yeast^9^. In human cells, H4R3 is targeted by various protein arginine methyltransferases (PRMTs) resulting in different methylation states. Specifically, PRMT1 catalyzes asymmetric dimethylation of H4R3 (H4R3me2a), PRMT5 deposits symmetric dimethylation to form H4R3me2s and PRMT7 also mediates H4R3me2s but mainly monomethylates this histone residue to form H4R3me1^15^. Interestingly, deregulation of these H4R3-associated PRMTs has been intimately linked to carcinogenesis, including CRC, through transcriptional control of genes implicated in diverse cellular processes, such as cell proliferation, DNA repair, and apoptosis^16,17^. Although we have previously reported an interplay between NAA40-mediated histone acetylation and H4R3 methylation in yeast^9^, this crosstalk has not yet been investigated in mammalian cells.

In this study, we show that NAA40 is significantly upregulated in primary CRC tissues and promotes CRC cell growth both in vitro and in xenograft tumor models. The results also indicate that in CRC cells NAA40 regulates H4R3me2s levels through transcriptional control of *PRMT5*, which subsequently modulates the expression of its cancer-associated target genes. Thus, our findings provide a link between NAA40 and colorectal carcinogenesis and suggest that this epigenetic enzyme could be a putative therapeutic target for CRC.

## Results

### NAA40 is highly expressed in CRC patients

**Fig. 1:**
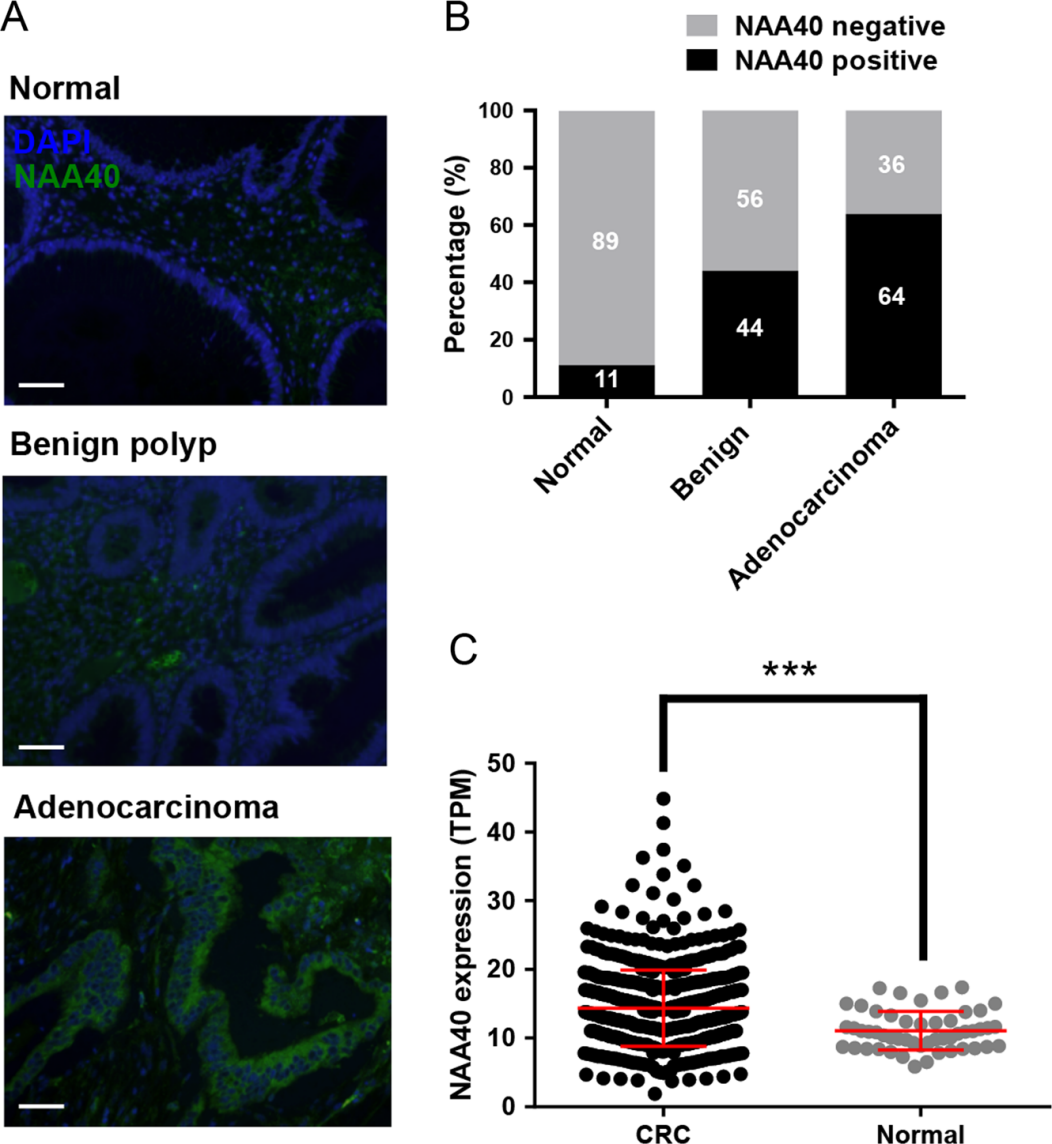
NAA40 is upregulated in CRC patient samples. **a** Representative images of immunofluorescence detection of NAA40 (green) on tissue microarrays containing a total of 318 normal and cancer colon tissues. Cell nuclei were visualized using DAPI staining (blue). Scale bar, 50 μm. **b** Graph showing the percentage of NAA40 positive and negative staining in normal adjacent tissues (*n* = 56), benign lesions (*n* = 30), and adenocarcinomas (*n* = 232). **c** Meta-analysis of *NAA40* mRNA expression levels extracted from the TCGA data portal in 647 colorectal cancer (CRC; COAD, *n* = 480 and READ, *n* = 167) and 51 normal patient samples (colon normal, *n* = 41 and rectum normal, *n* = 10). The Mann–Whitney test was used for the statistical analysis (***p < 0.001)

We have previously shown that NAA40 activity controls the survival of colon cancer cells^12^. To explore the clinical relevance of NAA40 expression in patients with CRC, we initially examined NAA40 protein levels on tissue microarrays harboring colon cancer tissues and adjacent normal specimens. Immunofluorescence analysis showed that NAA40 is increased in colon adenocarcinomas compared to benign lesions and normal colon specimens (Fig. 1a). In particular, the positive rates of NAA40 staining were 11%, 44%, and 64% for normal colon samples, benign tumors (polyps and adenomas), and adenocarcinomas, respectively (Fig. 1b). Consistently, meta-analysis of transcriptome data extracted from The Cancer Genome Atlas (TCGA) GDC Portal showed that *NAA40* mRNA levels in CRC patient tissues were significantly higher than those in normal colon tissues (Fig. 1c). However, we observed no significant correlation between the different tumor stages of colon adenocarcinoma and NAA40 expression at both the mRNA and protein levels based on the tumor, node, and metastasis classification obtained from the commercially available tissue microarrays and the TCGA network (Supplementary Figure S1). This may suggest that NAA40 upregulation occurs from the initial stages of malignant progression and is sustained along the different tumor stages. Overall, these results demonstrate that elevated NAA40 expression is a frequent event in colorectal carcinogenesis.

### Loss of NAA40 inhibits the growth of CRC cells in vitro

**Fig. 2:**
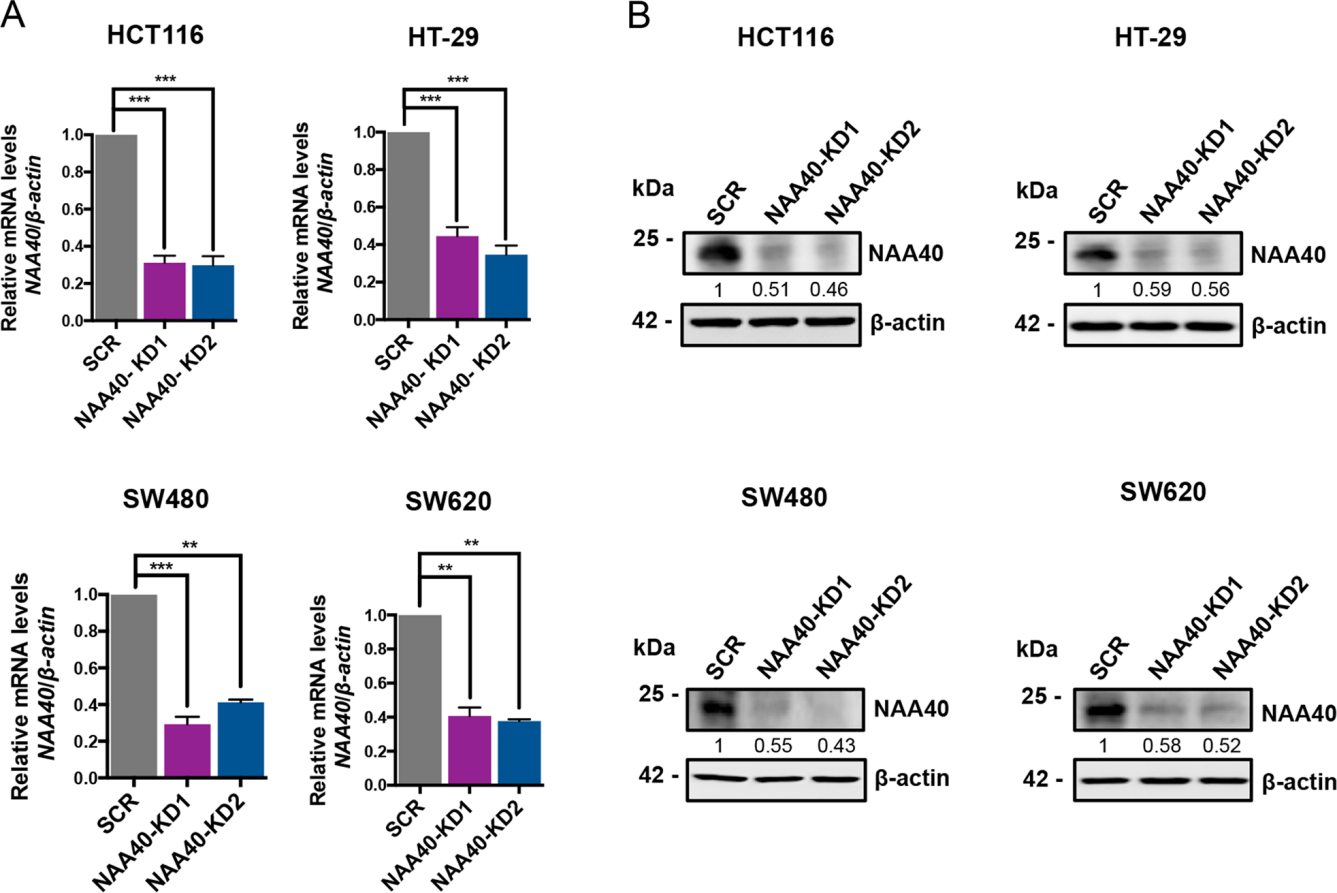
Generation of inducible NAA40-KD colon cancer cell lines. **a** Quantitative real-time PCR (qRT-PCR) analysis of *NAA40* mRNA levels normalized to *β-actin* performed in HCT116, HT-29, SW480, and SW620 cell lines expressing doxycycline-inducible scramble shRNA (SCR), or two different shRNA sequences targeting *NAA40* mRNA (NAA40-KD1 or NAA40-KD2). The relative values of *NAA40* transcripts represent the mean ± s.d. of three independent experiments. Unpaired two-tailed Student’s *t*-test was used (**p < 0.01, ***p < 0.001). **b** Western blot analysis of cell extracts derived from dox-treated SCR or NAA40-KD HCT116, HT-29, SW480, and SW620 cells using antibodies against NAA40 and β-actin as loading control. The numbers below each blot indicate densitometry analysis of NAA40 protein levels in NAA40-KD relative to SCR after normalization to the β-actin bands

To further evaluate the role of NAA40 in the context of CRC cell growth, we constructed an inducible system to downregulate NAA40 upon doxycycline (dox) treatment of HCT116, HT-29, SW480, and SW620 colon cancer cell lines. For this purpose we used two different lentivirus-based shRNA sequences (NAA40-KD1 and NAA40-KD2) targeting two distinct sites of *NAA40* mRNA. Dox administration in all engineered cell lines significantly reduced *NAA40* mRNA levels in the NAA40-KD1 and NAA40-KD2 cells compared to the negative scrambled control (SCR) cells as indicated by quantitative real-time PCR (qRT-PCR) (Fig. 2a). Accordingly, western blot analysis showed substantial decrease of NAA40 protein levels in all four cell lines tested upon dox-mediated induction of both NAA40 shRNAs as opposed to the induction of the control SCR shRNA (Fig. 2b).

**Fig. 3:**
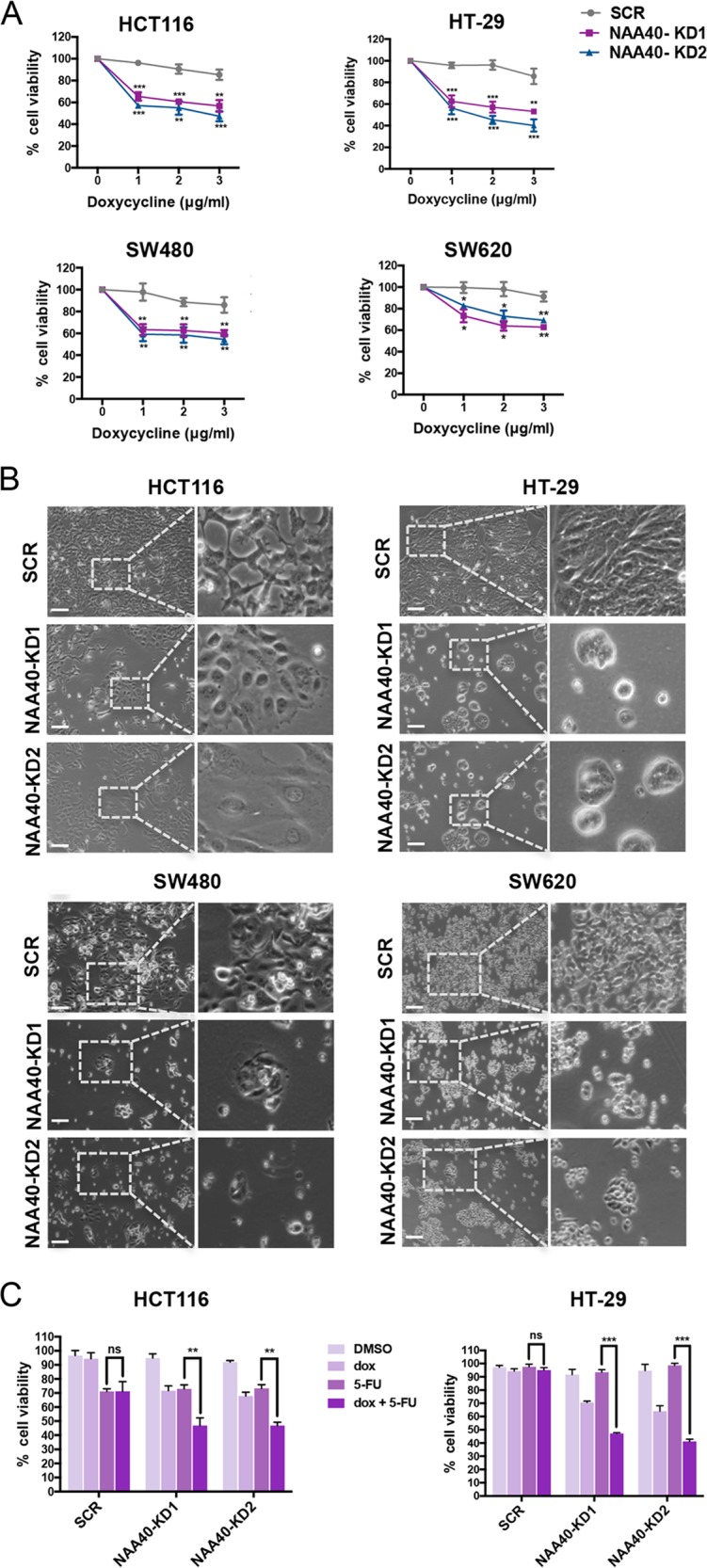
NAA40 knockdown reduces CRC cell growth in vitro. **a** MTT cell viability assay of SCR and NAA40-KD HCT116, HT-29, SW480, or SW620 cells incubated with various concentrations of dox. Cell viability is shown as a percentage relative to the corresponding dox untreated cells. The data are shown as mean ± s.d. of three replicates. Unpaired two-tailed Student’s *t*-test was used (*p < 0.05, **p < 0.01, ***p < 0.001 compared to SCR control cells). **b** Phase contrast microscopy of dox-inducible HCT116, HT-29, SW480, and SW620 engineered cell lines. Dashed rectangles indicate the zoomed-in areas shown in the right panels. Cells were captured in at least five fields of view (×100 magnification). The images are representative microscope fields from at least three independent replicates. Scale bar, 100 μm. **c** MTT assay of SCR and NAA40-KD HCT116 (left) and HT-29 (right) cells treated with DMSO, dox, 5-FU, or dox + 5-FU. Cell viability is presented as a percentage relative to the corresponding untreated cells. Error bars represent the mean ± s.d. of three replicates. Unpaired two-tailed Student’s *t*-test was used to compare 5-FU treatment alone to the treatment of 5-FU in combination with dox (ns = no significance, **p < 0.01, ***p < 0.001)

Next, to examine whether NAA40 depletion attenuates CRC cell growth in our cell line model, we used the MTT assay to measure cell viability. In agreement with our previous observations^12^, exposure of NAA40-KD HCT116, HT-29, SW480, and SW620 cells to different dox concentrations (1–3 μg/ml) exhibited significant loss of viability in contrast to the SCR control cells (Fig. 3a). Consistently, phase contrast microscopy showed that depletion of NAA40 impeded cell proliferation and resulted in morphological alterations, such as cellular rounding (Fig. 3b).

Although 5-fluorouracil (5-FU) is the most widely used chemotherapeutic agent for the treatment of CRC, resistance to 5-FU is an important reason for treatment failure^18^. However, previous evidence has shown that deregulation of various epigenetic mechanisms could impact on the effectiveness of 5-FU therapy in CRC^19^. Therefore, we next sought to investigate whether NAA40 controls the sensitivity of HCT116 and HT-29 cells to 5-FU treatment. We observed that sequential delivery of NAA40-KD cells with dox for 72 h followed by 5-FU for 24 h significantly reduces cell viability compared to the treatment of cells with 5-FU alone (Fig. 3c). This combinatorial effect of NAA40 knockdown followed by 5-FU treatment is especially apparent in HT-29 cells which are relatively resistant to 5-FU^20^. Notably, no differences were observed in cell viability when treating SCR cells with the two agents in combination as opposed to 5-FU alone (Fig. 3c). Taken together, our data show that lack of NAA40 suppresses in vitro growth of colon cancer cell lines and enhances the sensitivity of CRC cells to 5-FU.

### NAA40 depletion impairs CRC xenograft tumor growth

**Fig. 4:**
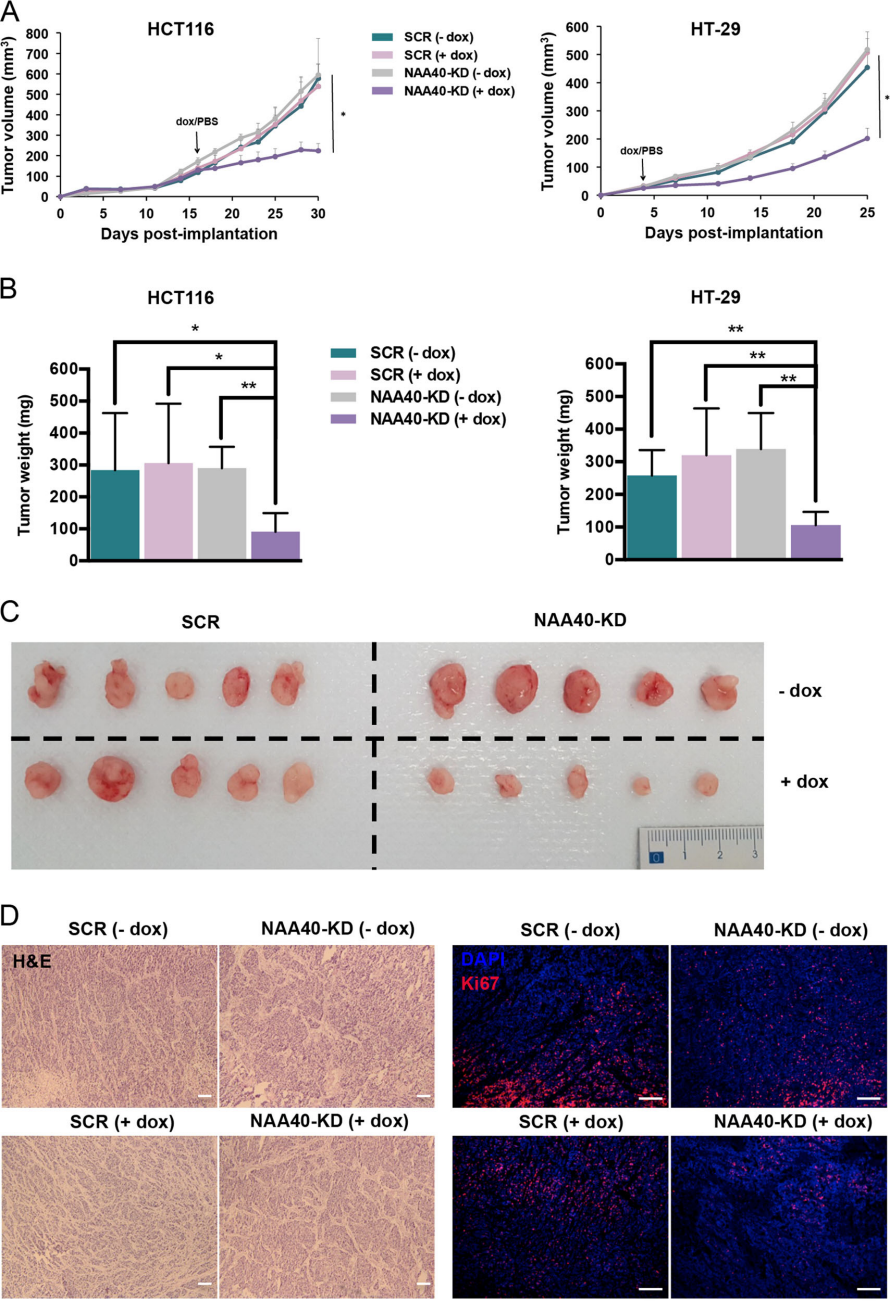
NAA40 depletion inhibits CRC cell growth in vivo. **a** The mean volume of tumors from the dox-treated (+dox) and PBS-treated (−dox) HCT116 (left) or HT-29 (right) xenografts harboring the conditionally induced SCR or NAA40-KD2 shRNAs. The tumor volume is shown as mean ± s.d. Unpaired two-tailed Student’s *t*-test was used (*p < 0.05). **b** At the end of the experiment in (**a**) mice were sacrificed and tumors were excised and weighted. Error bars represent mean ± s.d. Unpaired two-tailed Student’s *t*-test was used (*p < 0.05, **p < 0.01). **c** Representative tumors excised from SCR and NAA40-KD mouse xenografts after administration of dox (+dox) or PBS (−dox). **d** Representative hematoxylin & eosin (H&E) images from tumors derived from SCR and NAA40-KD mouse xenografts after administration of dox (+dox) or PBS (−dox). Scale bar, 200 μm. Representative immunofluorescence Ki67 staining (red) images from tumors derived from SCR and NAA40-KD mouse xenografts after administration of dox (+dox) or PBS (−dox). Cell nuclei were visualized using DAPI staining (blue). Scale bar, 100 μm

The effects of NAA40 deficiency on CRC cell proliferation in vitro prompted us to examine whether NAA40 regulates CRC tumor growth in vivo. To address this speculation, we subcutaneously injected HCT116 and HT-29 cells bearing dox-regulated lentiviral vectors expressing either SCR or NAA40-KD shRNAs into 6-week old CD1 nude immunodeficient mice. Notably, NAA40-KD xenograft tumors in mice treated with dox showed significant inhibition in growth relative to the NAA40-KD tumors in mice receiving PBS (-dox), as well as the SCR tumors in mice treated with either dox or PBS (Fig. 4a). At the end of the experiment, mice were sacrificed and tumors were excised. In agreement to effects observed with tumor volume, the weight of resected tumors from dox-treated NAA40-KD mice was reduced to ~33% of the weight in the control groups (Fig. 4b). The excised tumors representing the four treated groups were aligned for comparison (Fig. 4c). Histological hematoxylin and eosin (H&E) staining indicated no major morphological changes between tumors from all groups. Interestingly, immunofluorescence Ki67 staining revealed a significant decrease in the fraction of proliferating cancer cells upon NAA40 depletion (Fig. 4d). Our ex vivo analysis further confirmed that inducible NAA40 knockdown results in downregulation of NAA40 protein levels (Supplementary Figure S2), consistent with our in vitro data. Collectively, our findings indicate that NAA40 knockdown significantly decreases the growth of CRC xenografts in vivo.

### NAA40 stimulates PRMT5 expression in CRC cells

**Fig. 5:**
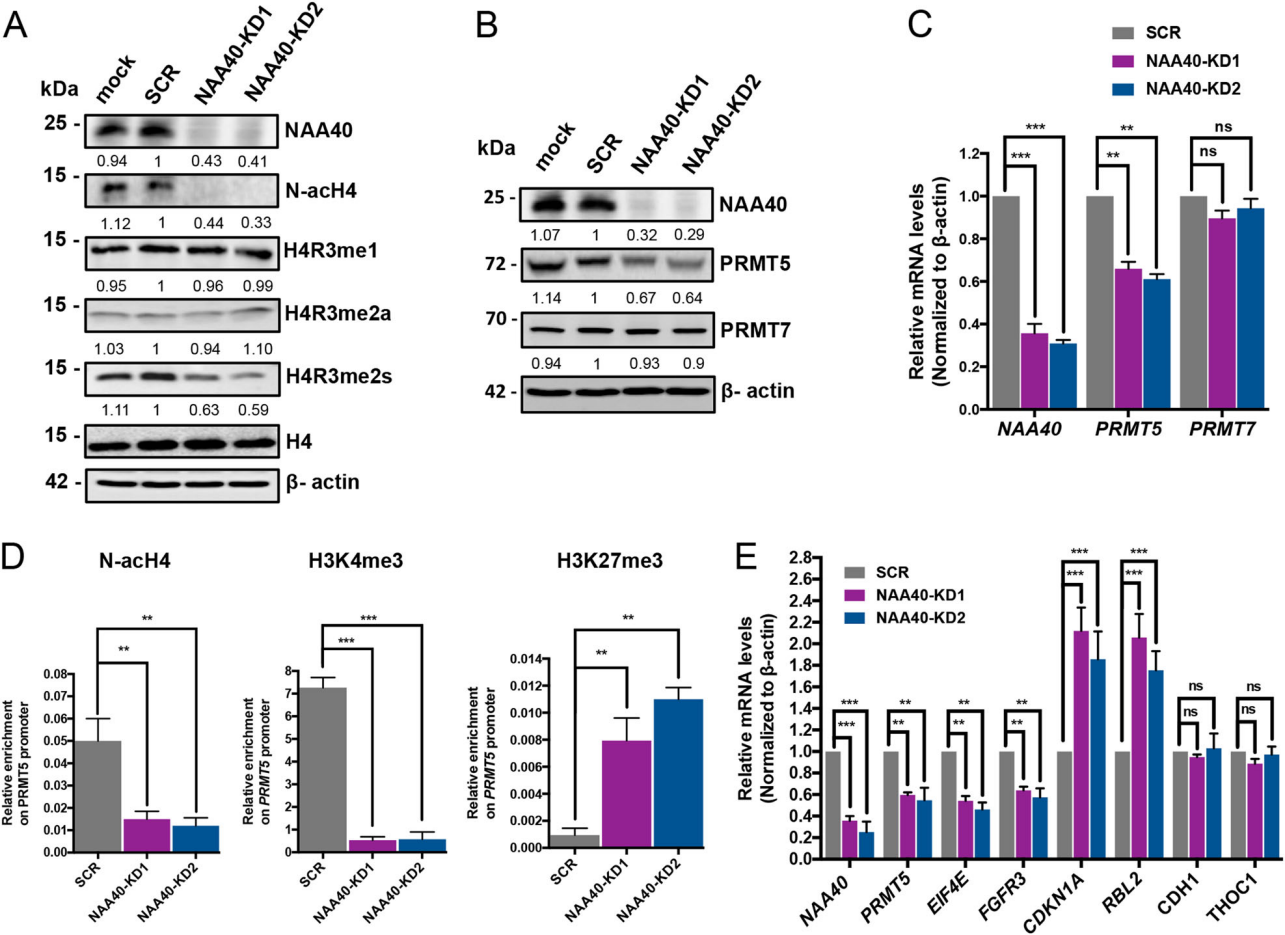
NAA40 regulates PRMT5 expression. **a** Western blot analysis of protein extracts from the indicated transiently transfected HCT116 cells using antibodies against NAA40, H4R3me1, H4R3me2a, H4R3me2s, total histone H4, and β-actin as loading control. The densitometry numbers below each blot define the normalized levels of H4R3me1, H4R3me2a, and H4R3me2s against H4 and of NAA40 against β-actin relative to SCR cells. **b** Western blot analysis of protein extracts from the indicated siRNA-treated cells using antibodies toward NAA40, PRMT5, PRMT7, and β-actin as loading control. The values below each blot were calculated by densitometry analysis of NAA40, PRMT5, and PRMT7 bands relative to SCR control after normalization with β-actin. **c** qRT-PCR analysis of mRNA levels of *NAA40*, *PRMT5*, and *PRMT7* normalized to *β-actin* in the indicated HCT116 cells. Error bars represent the mean ± s.d of three biological replicates. Unpaired two-tailed Student’s *t*-test was used (ns = no significance, **p < 0.01, ***p < 0.001). **d** ChIP assay monitoring the enrichment of N-acH4, H3K4me3, and H3K27me3 on the *PRMT5* gene promoter. The enrichment from each antibody was normalized to total histone H3. Results are shown as mean ± s.d. from two independent experiments. Unpaired two-tailed Student’s *t*-test was used (**p < 0.01, ***p < 0.001). **e** qRT-PCR analysis of mRNA levels in HCT116 cells for the indicated genes. The results were normalized to *β-actin* mRNA and represent the average from three independent experiments. Unpaired two-tailed Student’s *t*-test was used (ns = no significance, **p < 0.01, ***p < 0.001)

The above findings demonstrated a link between NAA40 expression and CRC cell growth. Therefore, we next sought to determine how NAA40 controls the viability of colon cancer cells. We have previously reported that in yeast cells NAA40-mediated N-terminal acetylation of histone H4 (N-acH4) controls the expression of a specific cohort of genes by regulating the deposition of the adjacent histone H4 arginine 3 asymmetric dimethylation (H4R3me2a) mark^8,9^. Therefore, we wondered if human NAA40 regulates gene expression and thereby cell growth by controlling the deposition of methyl marks at H4R3 in CRC cells. To test this hypothesis, we investigated the global cellular levels of H4R3me modifications in HCT116 cells depleted of NAA40. We found that H4 arginine 3 symmetric dimethylation (H4R3me2s) levels are notably decreased in the absence of NAA40 and of its mediated N-acH4 compared to the SCR and mock control cells. On the other hand, reduction of NAA40 expression did not influence the total levels of monomethylation (H4R3me1) or asymmetric dimethylation (H4R3me2a) at the third residue of histone H4 (Fig. 5a). Consistently, H4R3me2s levels were reduced upon NAA40 knockdown in SW480 and SW620 cells, while H4R3me1 and H4R3me2a levels remained unaffected (Supplementary Figures S3A and S4A).

Since the H4R3me2s mark is mainly deposited by the PRMT5 arginine methyltransferase, we then examined whether NAA40 knockdown in HCT116 cells affects the levels of this histone-modifying enzyme. We observed that PRMT5 protein levels are reduced in response to NAA40 knockdown as opposed to the SCR and mock controls. No significant differences were noted in PRMT7 protein levels, which is another enzyme mediating H4R3me2s (Fig. 5b). Moreover, loss of NAA40 decreased PRMT5 but not PRMT7 protein levels in SW480 and SW620 cells (Supplementary Figures S3B and S4B). Accordingly, PRMT5 protein levels are also reduced in NAA40-depleted xenograft tumors (Supplementary Figure S5). To validate this observation, we also examined the mRNA levels of *PRMT5* and *PRMT7* upon NAA40 depletion. Consistent with the above findings, NAA40 knockdown significantly reduced *PRMT5* but not *PRMT7* mRNA levels (Fig. 5c and Supplementary Figures S3C and S4C), suggesting that NAA40 controls *PRMT5* expression at the transcriptional level. In line with this, using chromatin immunoprecipitation (ChIP) assays we detected markedly reduced deposition of N-acH4 and of the transcriptionally activating H3K4me3 modification, while the repressive mark H3K27me3 was significantly enriched, at the *PRMT5* gene promoter in HCT116 cells depleted of NAA40 (Fig. 5d).

To substantiate the association between NAA40 and PRMT5 expression, we next sought to examine the expression of PRMT5 direct target genes upon NAA40 depletion. We focused our analysis on target genes whose control of expression by PRMT5 has been previously linked to CRC growth^21^. Accordingly, we found that *NAA40* reduction and subsequent downregulation of *PRMT5* results in decreased expression of Fibroblast Growth Factor Receptor 3 (*FGFR3*) and Eukaryotic Translation Initiation Factor 4E (*EIF4E*) oncogenes and derepression of RB Transcriptional Corepressor Like 2 (*RBL2*) and Cyclin Dependent Kinase Inhibitor 1 A (*CDKN1A*) tumor suppressors, which, respectively, are activated and repressed by PRMT5 in proliferating CRC cells (Fig. 5e and Supplementary Figures S3D and S4D). Conversely, there was no significant change in the expression of cadherin-1 (*CDH1*) and THO complex 1 (*THOC1*) control genes (Fig. 5e). Altogether, these results suggest that NAA40 promotes *PRMT5* transcriptional activation, which in turn influences the expression of vital cancer-associated genes in CRC cells.

### PRMT5 upregulation restores viability in NAA40-depleted CRC cell

**Fig. 6:**
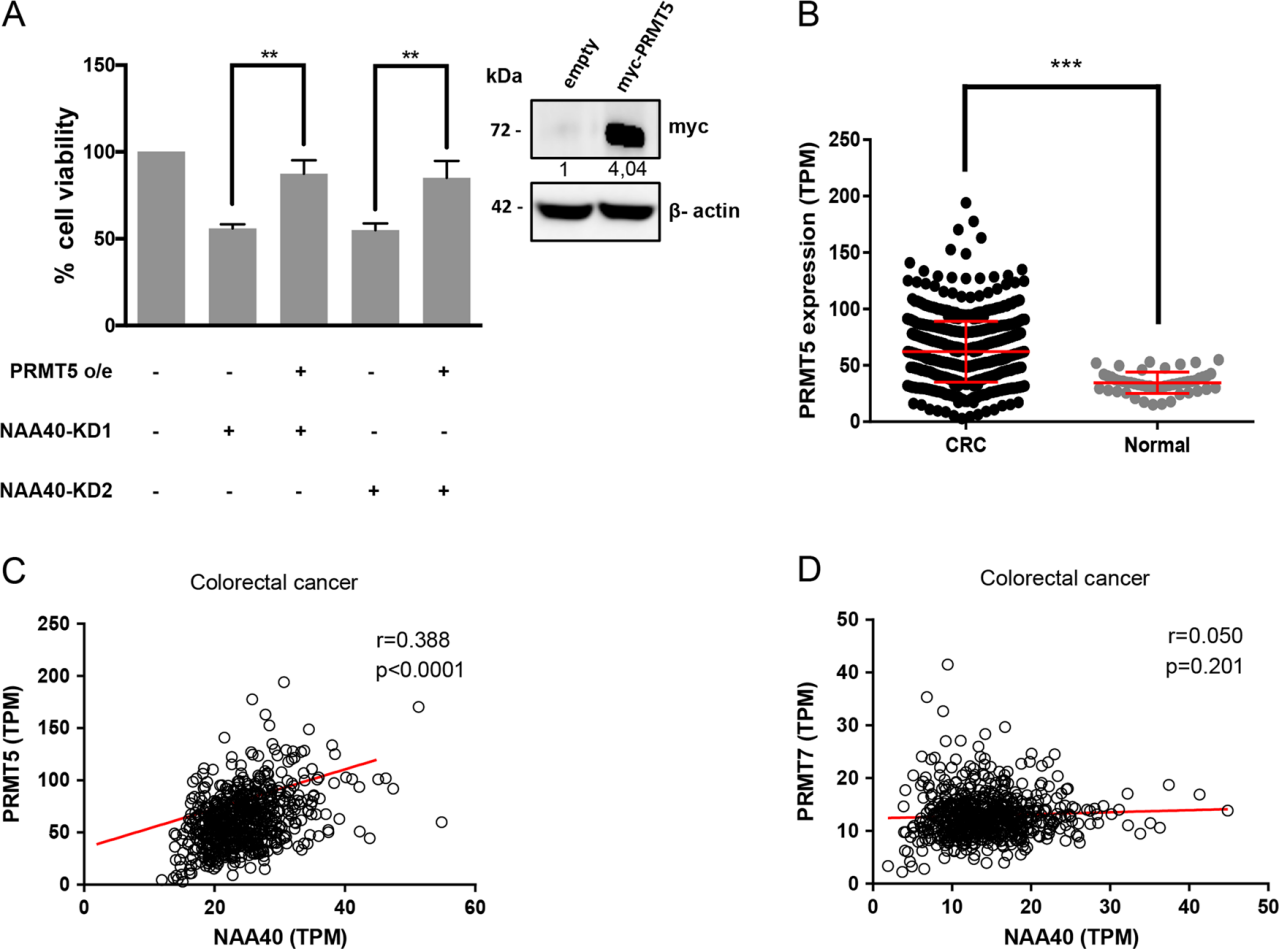
PRMT5 contributes to NAA40-dependent CRC cell growth. **a** MTT assay of HCT116 colon cancer cells expressing an empty vector (−) or a vector containing myc-tagged PRMT5 (+) in SCR (−) or NAA40-KD (+) conditions. Error bars represent the mean ± s.d of three biological replicates. Unpaired two-tailed Student’s *t*-test was used (**p < 0.01). Western blot analysis (right panel) was performed to detect ectopic expression of tagged PRMT5 using an antibody against myc. β-actin was used as a loading control. The values below the blot were determined by densitometry analysis. **b** Meta-analysis of *PRMT5* expression levels extracted from the TCGA data portal in 647 colorectal cancer (CRC) and 51 normal patient samples. The Mann–Whitney test was used for the statistical analysis (***p < 0.001). **c**, **d** Scatter plots illustrating the correlation between the expression of *NAA40* and *PRMT5* (**c**) or between *NAA40* and *PRMT7* (**d**) in 647 CRC patient samples extracted from the TCGA database. The red line indicates the linear regression slope. Statistical analysis was performed using Pearson’s rank correlation coefficient (*r*)

The data shown above indicate that NAA40 knockdown reduces PRMT5 expression in colon cancer cells. To investigate whether PRMT5 downregulation is important for the decreased CRC cell viability mediated by NAA40 depletion, we performed MTT assay in NAA40-knockdown cells that overexpressed PRMT5. Notably, ectopic expression of PRMT5 restored to a great extent the viability of HCT116 cells that were depleted of NAA40 (Fig. 6a), suggesting that PRMT5 contributes to NAA40-dependent CRC cell growth. In support of this relationship, meta-analysis of RNA-seq data from TCGA showed that *PRMT5* is significantly upregulated in CRC patient samples compared to the non-cancerous specimens, as previously seen for NAA40 (compare Figs. 6b to 1c). Furthermore, similarly to *NAA40*, *PRMT5* expression levels are also increased in all tumor stages in CRC patients (Supplementary Figure S6). More importantly, we found a significant positive correlation (*r* = 0.388, *p* < 0.0001) between the mRNA levels of *NAA40* and *PRMT5* in CRC patient samples (Fig. 6c). In contrast, there was no significant association (*r* = 0.050, *p* = 0.201) between *NAA40* and *PRMT7* mRNA levels in these CRC samples (Fig. 6d), consistent with the fact that NAA40 depletion does not affect the levels of *PRMT7* (Fig. 5c). Thus, overall these findings suggest that NAA40 facilitates survival of colon cancer cells partly through upregulation of PRMT5 expression.

## Discussion

Emerging evidence shows that impaired levels of epigenetic modifiers and their corresponding histone marks alter gene expression and affect cell growth leading to carcinogenesis. Recently, deregulation of the evolutionarily conserved NAA40 enzyme towards N-terminal acetylation on histone H4 has been implicated in different types of cancer^10–12^. Although we have previously linked NAA40 to CRC^12^ it remained unclear whether NAA40 contributes to colorectal oncogenesis. Interestingly, in the present study we show that NAA40 expression is significantly higher in patient-derived CRC primary tissues than in non-cancerous specimens. Additionally, our findings illustrate that conditional depletion of NAA40 blocks the growth of multiple CRC cell lines and inhibits xenograft tumor formation in mice. Moreover, we provide evidence showing that NAA40 depletion and loss of N-acH4 significantly reduce the global levels of the adjacent histone mark H4R3me2s through transcriptional repression of *PRMT5* which in turn alters the expression of its own cancer-associated target genes. Consistent with this link between NAA40 and PRMT5, we show that enforced PRMT5 expression partly restores the survival of NAA40-depleted cells and *NAA40* expression levels are positively correlated to those of *PRMT5* in CRC patient tissues. Taken together, we speculate that NAA40 is crucial for CRC cell growth by acting as a modulator of the PRMT5 oncogene.

Arginine methylation of H4R3 is known to either activate or silence gene expression depending on the symmetric or asymmetric configuration of its dimethylated state. In yeast cells, H4R3me2a is considered to mediate transcriptional silencing, while H4R3me2s is an activating mark^22^. Conversely in humans, H4R3me2a activates gene expression, whereas H4R3me2s is linked to transcriptional repression^23,24^. Intriguingly, our previous reports together with our findings here point toward an interplay of N-acH4 with the silencing versions of dimethylated H4R3 in yeast (H4R3me2a) and human (H4R3me2s) cells, respectively^9^ (Fig. 5a). Notably, a decrease in H4R3me2s levels upon loss of human NAA40 and N-acH4 has also been detected in lung cancer cells^10^. The current study is specifically focused on monitoring N-acH4 since we have previously demonstrated in yeast that NAA40 regulates H4R3me2a through histone H4 but not histone H2A^9^. However, future work could also explore the contribution of N-acH2A in NAA40-mediated effects but this would require the generation of new tools such as the development of a specific antibody against N-acH2A. For instance, it would be intriguing to investigate whether N-acH2A affects respectively the PRMT5-mediated deposition of H2AR3me2s since, in contrast to yeast, human histones H2A and H4 are identical^7,25^. Moreover, it would be interesting to explore whether H2A variants bearing the sequence recognized by NAA40, such as H2A.X, can also be N-terminally acetylated and influence colon cancer cell growth^26^. Additionally, in budding yeast, NAA40-mediated N-acH4 directly inhibits the activity of the HMT1 methyltransferase toward H4R3 thus, blocking the deposition of H4R3me2a and activating rDNA expression^9^. On the contrary, the current study reveals that N-acH4 in human cells indirectly cross-talks with H4R3me2s through NAA40-mediated transcriptional activation of *PRMT5* (Fig. 5). Consistent with this indirect crosstalk in human cells, a recent biochemical study has demonstrated that N-acH4 has no direct influence on the PRMT5 methyltransferase activity^27^. Interestingly, a direct antagonistic crosstalk between N-acH4 and H4 serine 1 phosphorylation (H4S1ph) in human lung cancer cells has been recently reported^10^. Whether this antagonistic relationship of N-acH4 and H4S1ph is also operative in CRC could be the focus of future investigations.

PRMT5 has been extensively characterized as an oncogene that epigenetically silences the expression of vital tumor suppressor genes^28–31^. Paradoxically, PRMT5 was recently shown to enhance the transcription of important oncogenes despite depositing silencing histone marks^21,25^. As a result, PRMT5 deficiency can either restore the expression of tumor suppressor genes or reduce the transcription of oncogenes to block cancer cell growth. Previous data show that PRMT5-mediated histone methylation directly controls the expression of certain cancer-related genes to promote colorectal oncogenesis^21^. Consistently, we show here that depletion of NAA40 and subsequent downregulation of PRMT5 leads to induction of the *RBL2* and *CDKN1A* tumor suppressor genes and repression of the *EIF4E* and *FGFR3* oncogenes (Fig. 5e). These data indicate that PRMT5 plays a key role in NAA40-mediated cancer cell growth. Although the evidence connecting NAA40 to PRMT5 transcriptional activation and downstream expression of its target genes are indirect, the presence of N-acH4 at the PRMT5 promoter (Fig. 5d) suggests that histone N-terminal acetylation may have a direct role in transcription. Finally, given the abundance of N-acH4 in the proteome^32^, we anticipate that PRMT5 is not the sole player facilitating NAA40-mediated survival of CRC cells. This is also supported by the fact that the viability of NAA40 knockdown CRC cells is only partially rescued by PRMT5 overexpression (Fig. 6a). Consequently, to fully comprehend the regulatory network of NAA40 in CRC, future analysis should seek to determine all genes that are transcriptionally controlled by this epigenetic modulator.

The crucial role of NAT enzymes in the development and progression of several human cancers including CRC highlights their significant clinical implication^33,34^. Nevertheless, with the exception of NAA80 that specifically targets actin^35^, all other NATs have broad substrate selectivity by acetylating a vast array of cellular proteins. Therefore, NAT-inhibitors may trigger numerous side effects. Since NAA40 catalytic activity is restricted toward histones H4 and H2A^26^, this raises a great promise for the development of specific NAA40 small-molecule inhibitors. The potential of NAA40 to serve as a target in anticancer therapy is also reinforced by the data here which show its ability to sensitize CRC cancer cells toward conventional chemotherapeutic drugs such as 5-FU (Fig. 3c). Of note, the representative images of survived dox-induced NAA40-silenced cells shown in Fig. 3b reveal notable alterations in cellular morphology compared to SCR cells suggesting that NAA40-KD cells may exhibit increased sensitivity to other chemotherapeutic agents as well. For example, NAA40 was previously shown to regulate the sensitivity of hepatocellular carcinoma cells to cisplatin^11^. Thus, these data suggest that inhibition of NAA40 could be used in combinatorial treatment regimens. However, prior to the usage of NAA40-specific pharmacological inhibitors for colon cancer treatment, further studies are required to determine the effects of NAA40 depletion in normal colonic cells.

Taken together, our findings strengthen the importance of NAA40 to maintain CRC cell growth. We show that NAA40 oncogenic properties stimulate the global levels of H4R3me2s by transcriptionally activating *PRMT5* methyltransferase which in turn modulates the expression of key downstream cancer-related genes. Hence, this study provides an understanding of how NAA40 deregulation contributes to colorectal carcinogenesis and proposes that NAA40 may serve as a therapeutic target and prognostic marker for CRC.

## Materials and methods

### Cell culture

The HCT116 cell line was kindly provided by Dr. Pantelis Hatzis (Biomedical Sciences Research Center ‘Alexander Fleming’) and the HT-29 (catalog no. HTB-38), SW480 (catalog no. CCL-228), and SW620 (catalog no. CCL-227) cell lines were purchased from ATCC. All CRC cell lines were cultured in McCoy’s 5a medium (Gibco, Invitrogen) supplemented with 10% fetal bovine serum (Gibco, Invitrogen) and 1% penicillin/streptomycin (Gibco, Invitrogen). The human embryonic kidney HEK-293 T (catalog no. CRL-3216) cell line was purchased from ATCC and was cultured in DMEM medium (Gibco, Invitrogen) supplemented with 10% fetal bovine serum and 1% antibiotic (penicillin/streptomycin). Cells were grown in a humidified atmosphere at 37 °C containing 5% CO_2_ and were routinely tested for mycoplasma contamination.

### Plasmid construction, lentivirus production, and infection

The annealed shSCR and shNAA40 sequences were introduced between *AgeI* and *EcoRI* restriction sites in the lentiviral Tet-pLKO-puro vector (Addgene plasmid 21915). The generated pLKO-Tet-On-shSCR (SCR), pLKO-Tet-On-shNAA40-1 (NAA40-KD1), and pLKO-Tet-On-shNAA40-2 (NAA40-KD2) constructs were verified by DNA sequencing. For lentiviral packaging, each of the recombinant vectors was co-transfected with the psPAX2 lentivirus packaging vector and the PMD2G lentivirus envelope plasmid in HEK-293 T cells by using X-tremeGENE 9 DNA transfection reagent (Roche) according to the manufacturer’s instructions. The obtained lentiviral particles were used to infect CRC cells in the presence of 10 μg/ml polybrene and the pool of efficiently transduced cells was selected with 2 μg/ml puromycin (Sigma-Aldrich). For the shRNA induction, CRC cells were treated with 1 μg/ml doxycycline hyclate (Sigma-Aldrich) for 96 h. The short hairpin RNA oligonucleotides used for plasmid construction were purchased from Integrated DNA Technologies (IDT) (Supplementary Table 1).

### Transient RNA interference

Non-targeted scramble siRNA (SCR) and two NAA40-specific siRNAs (NAA40-KD1 and NAA40-KD2) were purchased from GenePharma (Shanghai, China) (Supplementary Table 2). The NAA40 siRNA1 and NAA40 siRNA2 sequences were taken from Liu et al.^11^. HCT116 cells were seeded in antibiotic-free medium and grown to 30% confluence at the time of transfection. Subsequently, the cells were transiently transfected with 7.5 nM of NAA40 siRNA or the negative control using Lipofectamin RNAiMAX (Invitrogen) according to the manufacturer’s instructions. At 72 h post-transfection cells were subjected to downstream assays indicated in Figs. 5a–e and 6a.

### Immunofluorescence analysis in patient-derived tissues

The patient tissue microarrays were purchased from Abcam (ab178122, ab178131, and ab178132). The slides were heated at 60 °C for 30 min and then permeabilised at RT in 4% paraformaldehyde for 10 min. After blocking in PBG (0.2% cold water fish gelatin, 0.5% BSA in 1X PBS) for 45 min, the tissues were incubated with the NAA40 antibody (1:100; ab106408, Abcam) overnight at 4 °C. Slides were stained with anti-rabbit FITC-conjugated secondary antibody (1:500; 711-095-152, Jackson ImmunoResearch) for 1 h at RT. Nuclei were visualized with DAPI (Dako). Immunofluorescence images were acquired using a ZeissAxio Observer.A1 microscope.

### Extraction of CRC datasets

CRC data were extracted from TCGA datasets TCGA-COAD (colon adenocarcinoma, *n* = 480) and TCGA-READ (rectum adenocarcinoma, *n* = 167) and represent only untreated primary tumors. Normal solid colorectal tissue data were also extracted from the same datasets (colon normal, *n* = 41 and rectum normal, *n* = 10). Patients who received neo-adjuvant therapy were excluded from the study. In specific, we extracted “level 3” mRNA-seq expression data of the genes of interest (*NAA40*, *PRMT5*, *PRMT7*), along with the corresponding patient clinical information from the Genomic Data Commons (GDC) Data Portal of TCGA program (https://portal.gdc.cancer.gov/). Total raw read counts per gene were divided by the gene’s maximum transcript length to represent a coverage depth estimate. Coverage estimates were then scaled to sum to a total depth of 1e6 per sample and interpreted as transcripts per million after adding a 0.01 offset to remove the zero counts from calculations.

### MTT assay

To assess cell viability, CRC cells were seeded in a 96-well plate and incubated with 1–3 μg/μl doxycycline for 96 h. For the experiments shown in Fig. 3c, 5-FU (Sigma), or DMSO vehicle/control (Gibco, Invitrogen) were added at the concentration of 15 μM (HCT116) and 200 μM (HT-29) for 24 h following pre-incubation of SCR, NAA40-KD1, and NAA40-KD2 stable cells with doxycycline for 72 h. Relating to the experiments shown in Fig. 6a, 24 h siRNA-treated HCT116 cells were transfected with 1 μg of pcDNA-myc-empty or pcDNA-myc-PRMT5 expression vectors kindly provided by Prof. Naoya Fujita (Cancer Chemotherapy Centre of JFCR) using X-tremeGene9 reagent (Sigma-Aldrich) for 48 h. At the end of each treatment, 1 mg/ml MTT dye (Invitrogen) was added to each well and then cells were placed at 37 °C for 3 h. The formazan product was solubilized in DMSO and the plate was shaken for 20 min in dark to dissolve formazan crystals. The absorbance was read at 570 nm by using Perkin Elmer Wallac Victor 1420-002 Multilabel Counter.

### Tumor xenografts in nude mice

The xenograft studies were performed at the animal facility of the Cyprus Institute of Neurology and Genetics under Dr. Papageorgis’ animal project license (CY/EXP/PR.L1/2016) issued and approved by the Cyprus Veterinary Services that is the Cyprus national authority for monitoring animal research for all academic institutions according to the regulations contained in the Cyprus Law N.55 (I)/2013 and the EU Directive 2010/63/EU. A total of 2.5 × 10^6^ NAA40-KD2 or SCR control HCT116 and 2.5 × 10^6^ NAA40-KD2 or SCR control HT-29 cells were suspended in 40 μl serum-free McCoy’s 5a medium and inoculated subcutaneously in the flank of 6-week old male CD1 nude immunodeficient mice. Once the tumors reached an average size of about 100 mm^3^ (HCT116) or 50 mm^3^ (HT-29), all groups were size-matched (*n* = 7) and mice received daily 0.2 ml dox (100 mg/kg) or PBS (-dox/control) through oral gavage. Throughout the experiment, tumor volume was measured twice a week using a digital caliper and calculated using the volume of an ellipsoid and assuming that the third dimension, *z*, is equal to$\sqrt {xy}$. Therefore, the volume was given by the equation: $V = \frac{{4\pi }}{3}\frac{{(xyz)}}{8}$. At the end of each study (30th day for HCT116 and 25th day for HT-29 xenografts) mice were euthanized and tumors were excised, weighted, and subjected to histological analysis.

### Histological analysis of xenograft tumors

Primary tumors isolated from mice inoculated with either HCT116 or HT-29 CRC cells (NAA40-KD2 or SCR control, as described above), were fixed in 4% parafolmaldehyde and embedded in paraffin. Tissue sections (10µm-thick) were performed using an SRM200 microtome (Sakura), followed by staining with hematoxylin and eosin (H&E) using standard protocols, as previously described^36^. Immunohistochemical detection of mitotic cells, NAA40 and PRMT5 protein levels was performed by staining sections with anti-Ki67 (1:50; ab15580, Abcam), rabbit anti-NAA40 (1:100; ab106408, Abcam), and rabbit anti-PRMT5 (1:100; cat. 07-405, Millipore) antibodies, respectively, followed by FITC-conjugated secondary antibodies (1:400; 711-095-152, Jackson ImmunoResearch). All nuclei were stained with DAPI (Dako). Immunofluorescence microscopy images were obtained using a BX53 (Olympus) fluorescence microscope.

### RNA extraction and qRT-PCR

Total RNA was extracted using the RNeasy Mini kit (Qiagen) according to the manufacturer’s instructions and was then treated with DNAse using the TURBO DNAse kit (Ambion). An amount of 0.5 μg total RNA was then reverse transcribed to complementary DNA using the PrimeScript RT reagent kit (Takara) with random primers. qRT-PCR was carried out using KAPA SYBR Green (SYBR Green Fast qPCR Master Mix) and the Biorad CFX96 Real-Time System. Expression data were normalized to the mRNA levels of the *β-actin* housekeeping gene and calculated using the 2^−ΔΔCt^ method. Primer sequences were obtained from IDT (Supplementary Table 3).

### Western blot analysis

Protein extracts were isolated using Lysis Buffer (50 mΜ Tris-HCL pH 8, 3 mM EDTA, 100 mM NaCL, 1% Triton-X-100, 10% glycerol, 0.5 mM PMSF, and Sigma protease inhibitor cocktail) and the protein concentration was quantified by Bradford assay (BioRad). Twenty to fifty micrograms (20–50 μg) of protein extract was separated on SDS-PAGE and then transferred to a nitrocellulose membrane (GE Healthcare). After blocking with 5% TBS-T/BSA for 1 h at RT, the membranes were incubated with the primary antibodies overnight at 4 °C. The antibodies we used in this study were: PRMT5 (1:5000; cat. 07-405, Millipore), PRMT7 (1:2000; cat. 07-639, Millipore), H4 (1:1000; cat. 05-858, Millipore), β-actin (1:1000; sc-1616-R, Santa Cruz), H4R3me1 (1:1000; ab17339, Abcam), H4R3me2a^9^, H4R3me2s (1:1000; ab5823, Abcam), and c-myc (1:2000; sc-764, Abcam). For efficient NAA40 detection, whole cell extracts were resuspended in a tenfold volume of SDS loading buffer (50 mM Tris-HCL pH 6.8, 2% SDS, 10% glycerol, 1% β-mercaptoethanol, 12.5 mM EDTA, and 0.02% bromophenol blue) and alternatively boiled and chilled three times to disrupt cell membranes. The polyclonal antibody against NAA40 (1:1000) was kindly provided by Dr. Qiwei Zhai^11^. The rabbit polyclonal N-acH4 antibody was raised against the ac-NH-SGRGKGGKGLGKC antigen as previously described^12^. For secondary antibody, a Horseradish peroxide (HRP)-conjugated goat anti-rabbit IgG (Thermo Scientific) was used at a dilution 1:30000. Bands were detected by the enhanced chemiluminescence system (BioRad) and analyzed through densitometry using Image J analysis software (NJH). The intensity values were normalized against β-actin and are expressed relative to the SCR control.

### ChIP assay

HCT116 transfected cells were first fixed in 1% formaldehyde and quenched with 125 mM glycine. After the cells were lysed in SDS lysis buffer (1% SDS, 10 mM EDTA, 50 mM Tris-HCL pH 8, and protease inhibitor cocktail), the DNA was sheared by sonication in a Bioruptor (Diagenode). The chromatin was diluted 10-fold in IP buffer (1% Triton-X-100, 2 mM EDTA, 50 mM Tris-HCL pH 8, 150 mM NaCl, and protease inhibitor cocktail) followed by 1 h preclearing using Protein A sepharose beads (GE Healthcare) at RT. After incubation with 1 μg of antibodies against N-acH4, H3K4me3 (ab8580, Abcam), H3K27me3 (39156, Active Motif), H3 (ab1791, Abcam), or IgG (Biogenesis 5180-2104) for 1 h at 4 °C, 50% slurry protein A beads blocked in salmon sperm DNA were added and incubated overnight at 4 °C. Following washing steps, the immunoprecipitated chromatin was eluted in freshly prepared elution buffer (1% SDS and 0.1 M NaHCO3) and reverse cross-linked using 200 mM NaCl containing 0.5 μg/μl RNase (Roche) at 65 °C overnight. The samples were purified using the QIAquick PCR purification kit (QIAGEN) and analyzed with qRT-PCR using primer sequences of the *PRMT5* promoter region (Supplementary Table 3).

### Statistical analysis

All presented data are the mean ± s.d. of at least three independent experiments. Statistical analysis was carried out using GraphPad Prism (v.6.01, La Jolla, CA). Comparisons between groups were performed using unpaired Student’s *t*-test unless otherwise stated in the figure legend. Differences with *p < 0.05 were considered to be statistically significant.

## Supplementary information

Supplementary Figure S1

Supplementary Figure S2

Supplementary Figure S3

Supplementary Figure S4

Supplementary Figure S5

Supplementary Tables

Supplementary Figure S6

Supplemental Figure legends

## References

[1] Bannister AJ, Kouzarides T (2011). Regulation of chromatin by histone modifications. Cell Res..

[2] Kebede AF, Schneider R, Daujat S (2015). Novel types and sites of histone modifications emerge as players in the transcriptional regulation contest. FEBS J..

[3] Lawrence M, Daujat S, Schneider R (2016). Lateral thinking: how histone modifications regulate gene expression. Trends Genet..

[4] Demetriadou C, Kirmizis A (2017). Histone acetyltransferases in cancer: guardians or hazards?. Crit. Rev. Oncog..

[5] Di Martile M, Del Bufalo D, Trisciuoglio D (2016). The multifaceted role of lysine acetylation in cancer: prognostic biomarker and therapeutic target. Oncotarget.

[6] Aksnes H, Drazic A, Marie M, Arnesen T (2016). First things first: vital protein marks by n-terminal acetyltransferases. Trends Biochem. Sci..

[7] Hole K (2011). The human N-alpha-acetyltransferase 40 (hNaa40p/hNatD) is conserved from yeast and N-terminally acetylates histones H2A and H4. PLoS One.

[8] Molina-Serrano D (2016). Loss of Nat4 and its associated histone H4 N-terminal acetylation mediates calorie restriction-induced longevity. EMBO Rep..

[9] Schiza V, Molina-Serrano D, Kyriakou D, Hadjiantoniou A, Kirmizis A (2013). N-alpha-terminal acetylation of histone H4 regulates arginine methylation and ribosomal DNA silencing. PLoS Genet..

[10] Ju J (2017). NatD promotes lung cancer progression by preventing histone H4 serine phosphorylation to activate Slug expression. Nat. Commun..

[11] Liu Z (2009). Patt1, a novel protein acetyltransferase that is highly expressed in liver and downregulated in hepatocellular carcinoma, enhances apoptosis of hepatoma cells. Int. J. Biochem. Cell. Biol..

[12] Pavlou D, Kirmizis A (2016). Depletion of histone N-terminal-acetyltransferase Naa40 induces p53-independent apoptosis in colorectal cancer cells via the mitochondrial pathway. Apoptosis.

[13] Molina-Serrano D, Schiza V, Kirmizis A (2013). Cross-talk among epigenetic modifications: lessons from histone arginine methylation. Biochem. Soc. Trans..

[14] Zhang T, Cooper S, Brockdorff N (2015). The interplay of histone modifications - writers that read. EMBO Rep..

[15] Blanc RS, Richard S (2017). Arginine methylation: the coming of age. Mol. Cell.

[16] Poulard C, Corbo L, Le Romancer M (2016). Protein arginine methylation/demethylation and cancer. Oncotarget.

[17] Yang Y, Bedford MT (2013). Protein arginine methyltransferases and cancer. Nat. Rev. Cancer.

[18] Hammond WA, Swaika A, Mody K (2016). Pharmacologic resistance in colorectal cancer: a review. Ther. Adv. Med. Oncol..

[19] Du C (2017). 5-Fluorouracil targets histone acetyltransferases p300/CBP in the treatment of colorectal cancer. Cancer Lett..

[20] Zhang W (2003). Apoptotic response to 5-fluorouracil treatment is mediated by reduced polyamines, non-autocrine Fas ligand and induced tumor necrosis factor receptor 2. Cancer Biol. Ther..

[21] Zhang B (2015). Targeting protein arginine methyltransferase 5 inhibits colorectal cancer growth by decreasing arginine methylation of eIF4E and FGFR3. Oncotarget.

[22] Low JK, Wilkins MR (2012). Protein arginine methylation in Saccharomyces cerevisiae. FEBS J..

[23] Wang, H. et al. Methylation of histone H4 at arginine 3 facilitating transcriptional activation by nuclear hormone receptor. *Science* **293**, 853–857 (2001). 10.1126/science.1060781 11387442

[24] Zhao Q (2009). PRMT5-mediated methylation of histone H4R3 recruits DNMT3A, coupling histone and DNA methylation in gene silencing. Nat. Struct. Mol. Biol..

[25] Deng X (2017). Protein arginine methyltransferase 5 functions as an epigenetic activator of the androgen receptor to promote prostate cancer cell growth. Oncogene.

[26] Magin RS, Liszczak GP, Marmorstein R (2015). The molecular basis for histone H4- and H2A-specific amino-terminal acetylation by NatD. Structure.

[27] Fulton MD, Zhang J, He M, Ho MC, Zheng YG (2017). Intricate effects of alpha-amino and lysine modifications on arginine methylation of the N-terminal tail of histone H4. Biochemistry.

[28] Chung J (2013). Protein arginine methyltransferase 5 (PRMT5) inhibition induces lymphoma cell death through reactivation of the retinoblastoma tumor suppressor pathway and polycomb repressor complex 2 (PRC2) silencing. J. Biol. Chem..

[29] Kaushik S (2018). Genetic deletion or small-molecule inhibition of the arginine methyltransferase PRMT5 exhibit anti-tumoral activity in mouse models of MLL-rearranged AML. Leukemia.

[30] Tae S (2011). Bromodomain protein 7 interacts with PRMT5 and PRC2, and is involved in transcriptional repression of their target genes. Nucleic Acids Res..

[31] Wang L, Pal S, Sif S (2008). Protein arginine methyltransferase 5 suppresses the transcription of the RB family of tumor suppressors in leukemia and lymphoma cells. Mol. Cell. Biol..

[32] Tweedie-Cullen RY (2012). Identification of combinatorial patterns of post-translational modifications on individual histones in the mouse brain. PLoS One.

[33] Kalvik TV, Arnesen T (2013). Protein N-terminal acetyltransferases in cancer. Oncogene.

[34] Ren T (2008). Generation of novel monoclonal antibodies and their application for detecting ARD1 expression in colorectal cancer. Cancer Lett..

[35] Drazic A (2018). NAA80 is actin’s N-terminal acetyltransferase and regulates cytoskeleton assembly and cell motility. Proc. Natl Acad. Sci. USA.

[36] Papageorgis P (2015). Targeting IL13Ralpha2 activates STAT6-TP63 pathway to suppress breast cancer lung metastasis. Breast Cancer Res..

