# Supplementary figures and images for: NAA40 contributes to colorectal cancer growth by controlling PRMT5 expression
Source: Cell Death Dis. 2019 Mar 11;10(3):236. doi: 10.1038/s41419-019-1487-3 (PMC6411749; doi:10.1038/s41419-019-1487-3)

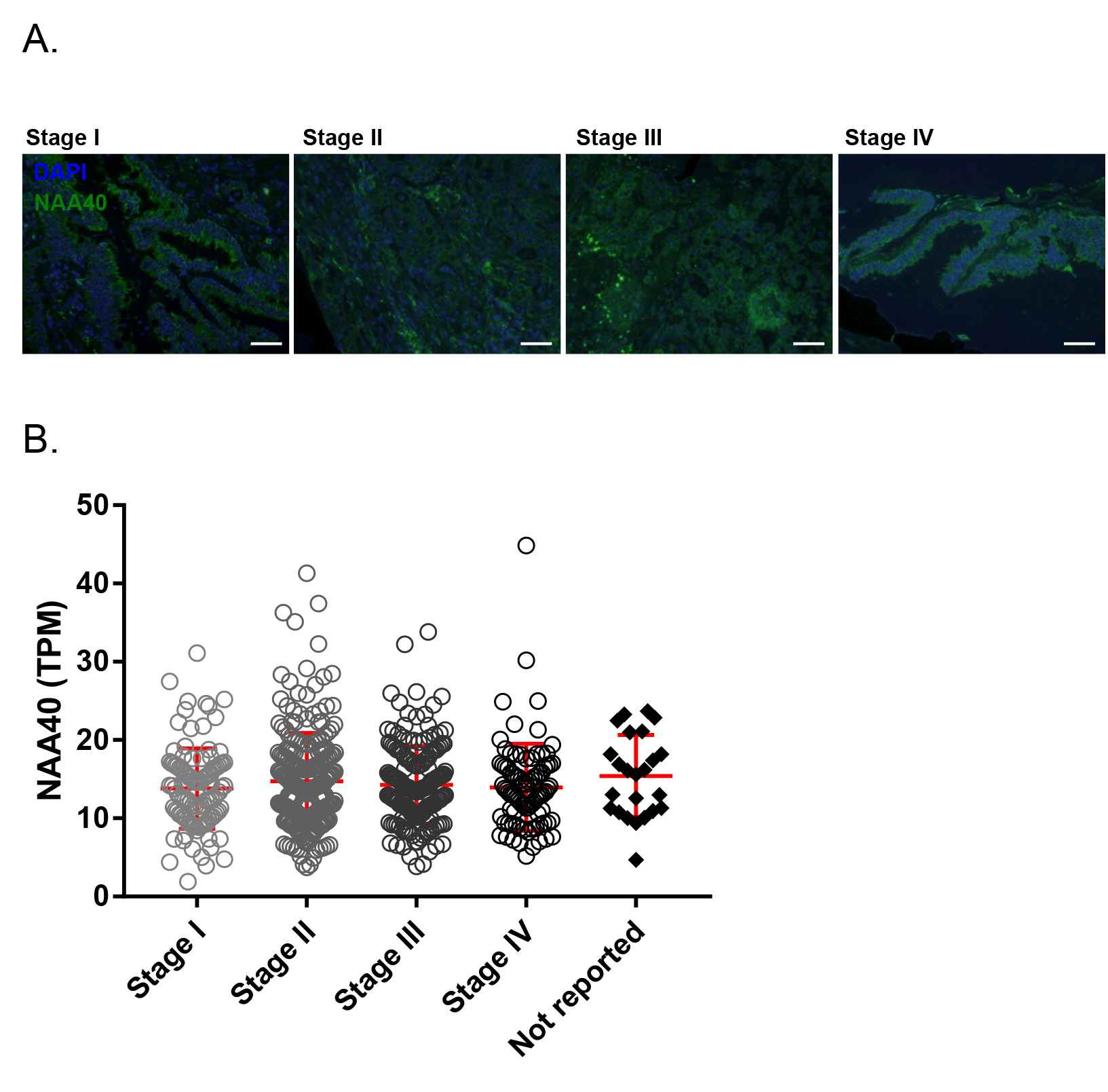

Supplement: Supplementary file 1 — Supplementary Figure S1 [file 41419_2019_1487_MOESM1_ESM.tif]

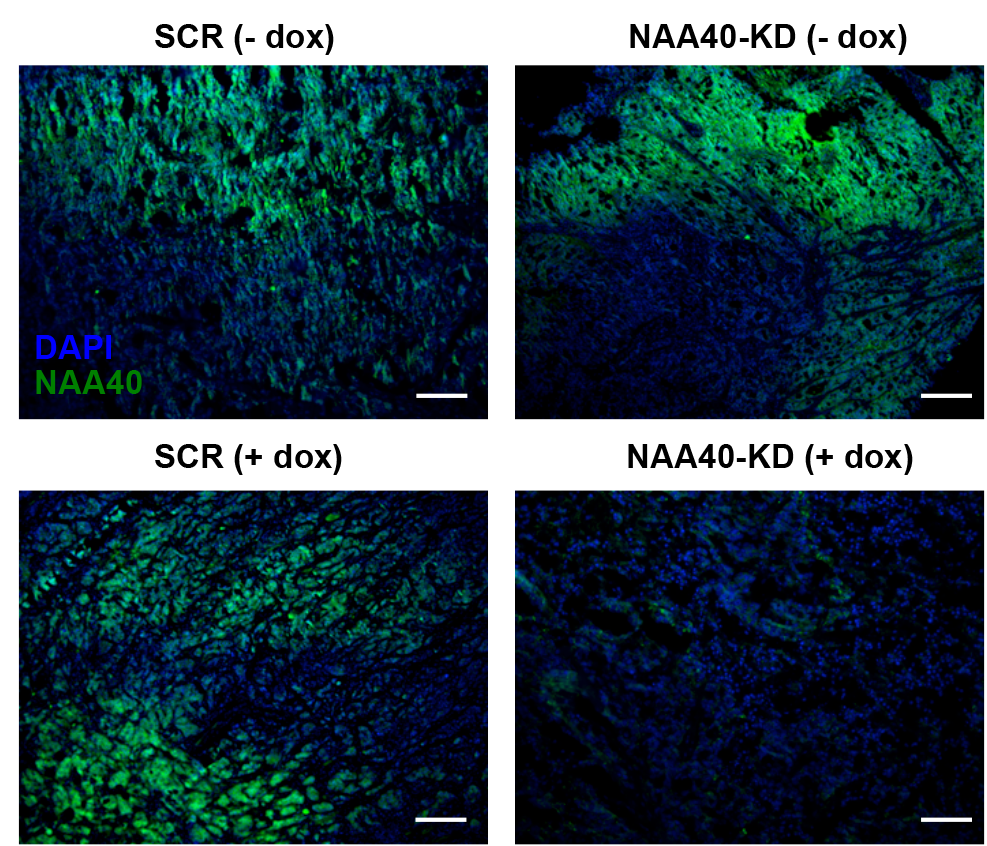

Supplement: Supplementary file 2 — Supplementary Figure S2 [file 41419_2019_1487_MOESM2_ESM.tif]

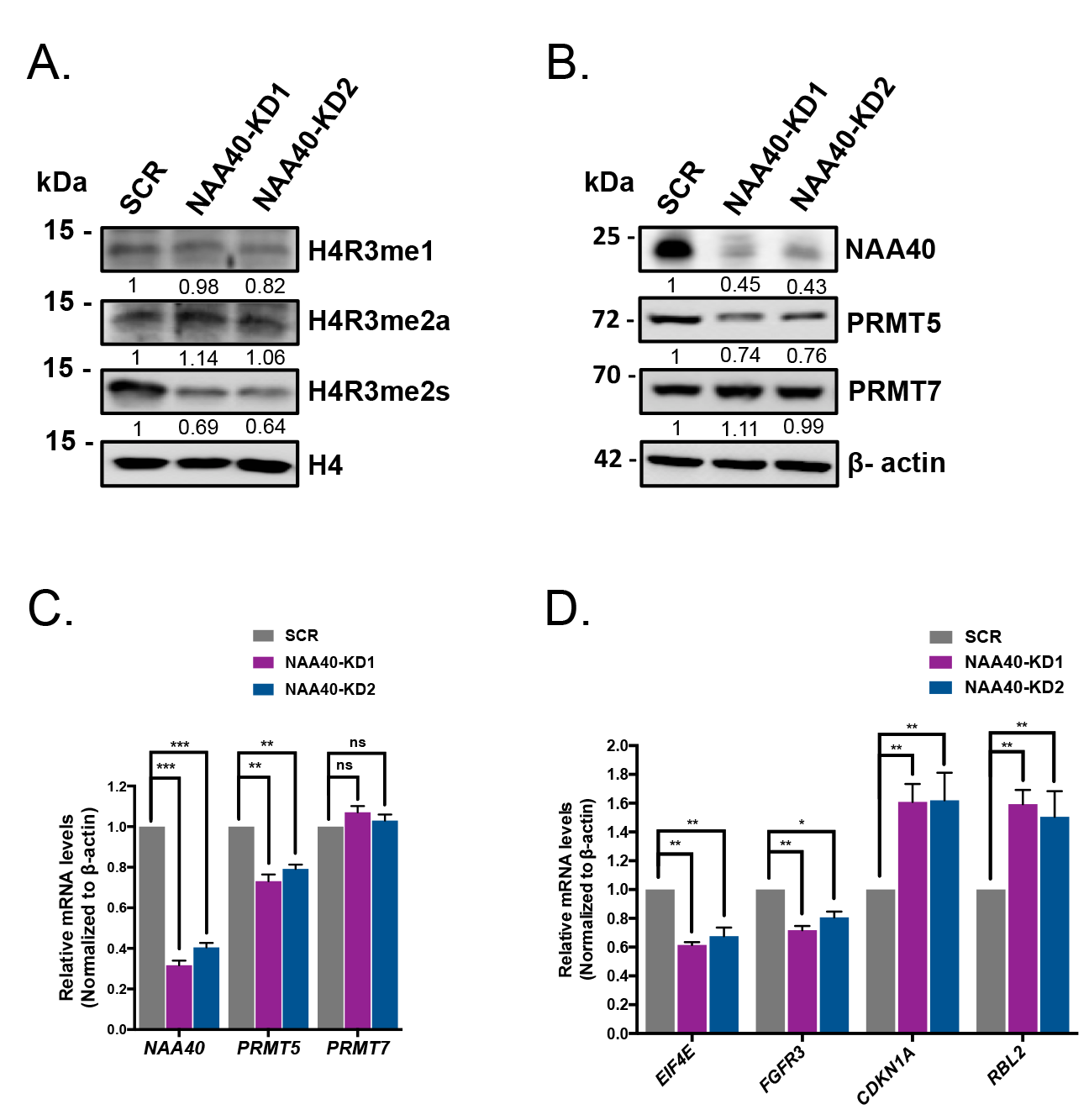

Supplement: Supplementary file 3 — Supplementary Figure S3 [file 41419_2019_1487_MOESM3_ESM.tif]

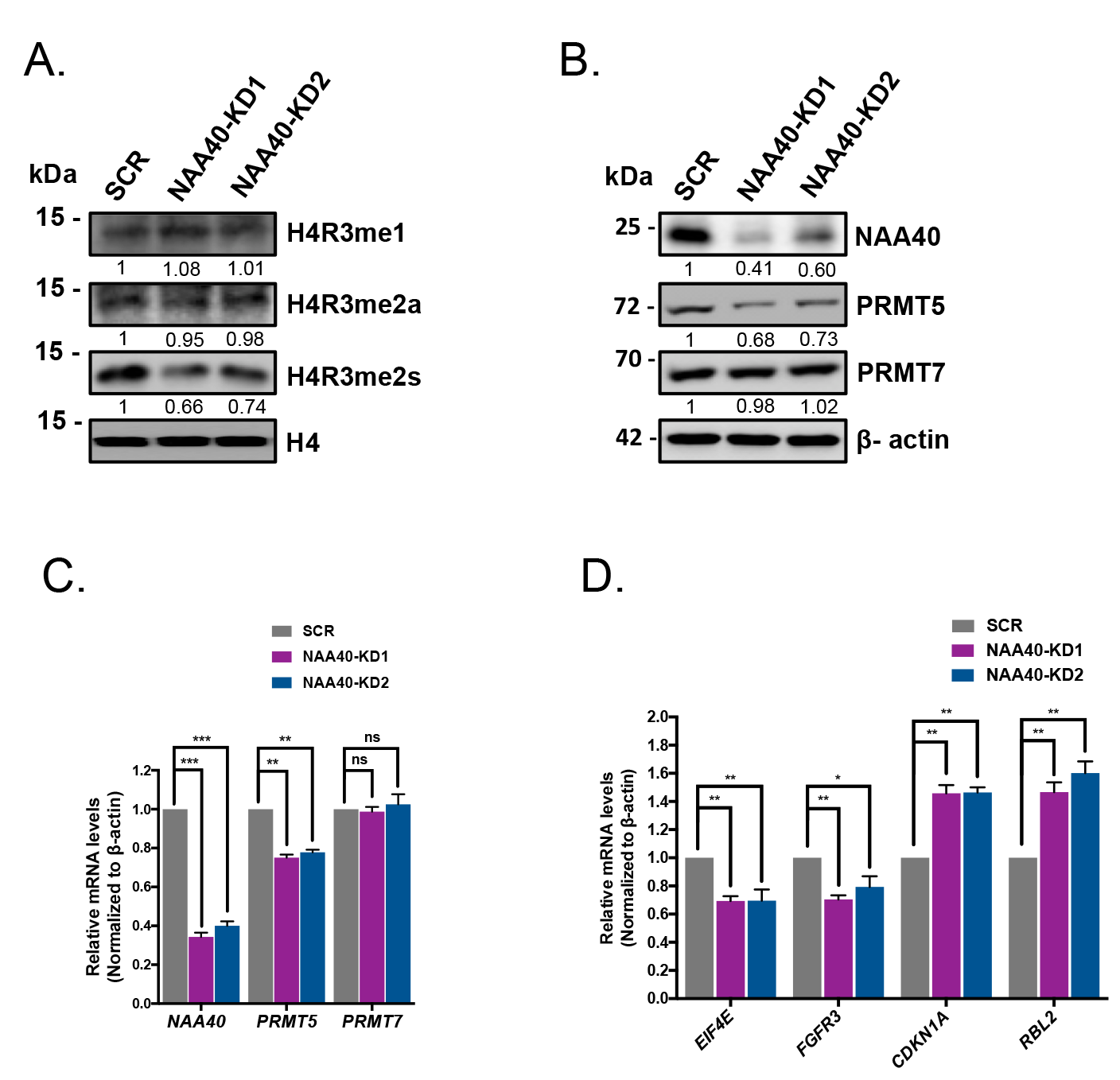

Supplement: Supplementary file 4 — Supplementary Figure S4 [file 41419_2019_1487_MOESM4_ESM.tif]

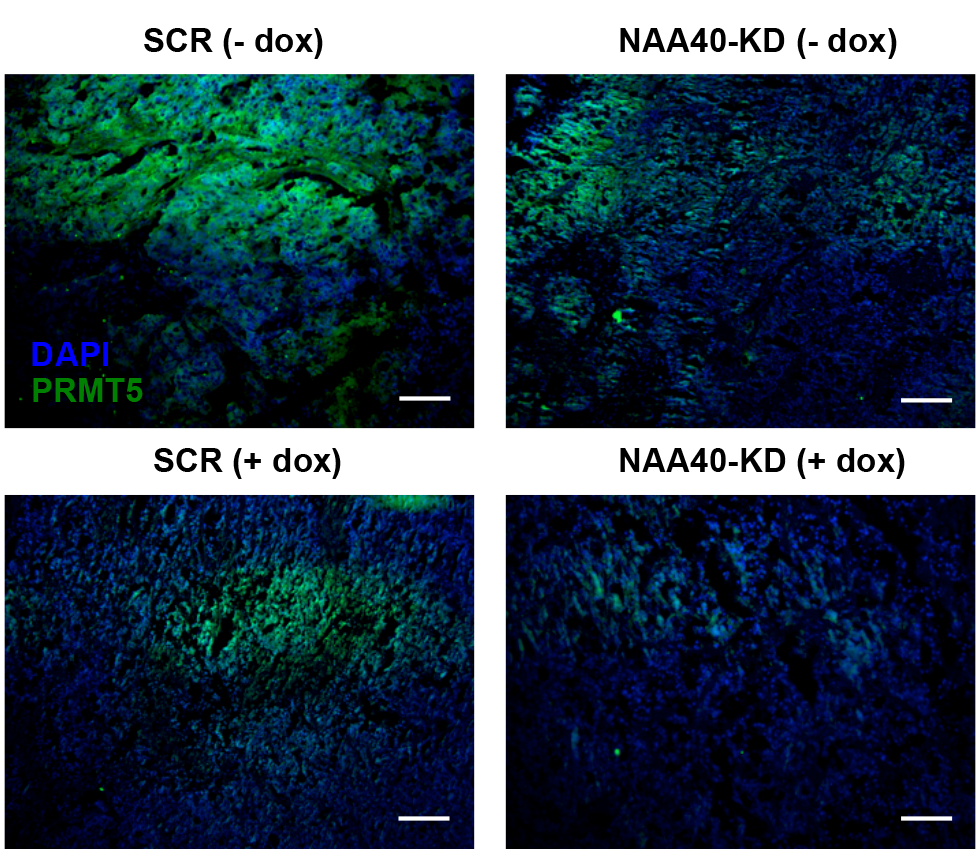

Supplement: Supplementary file 5 — Supplementary Figure S5 [file 41419_2019_1487_MOESM5_ESM.tif]

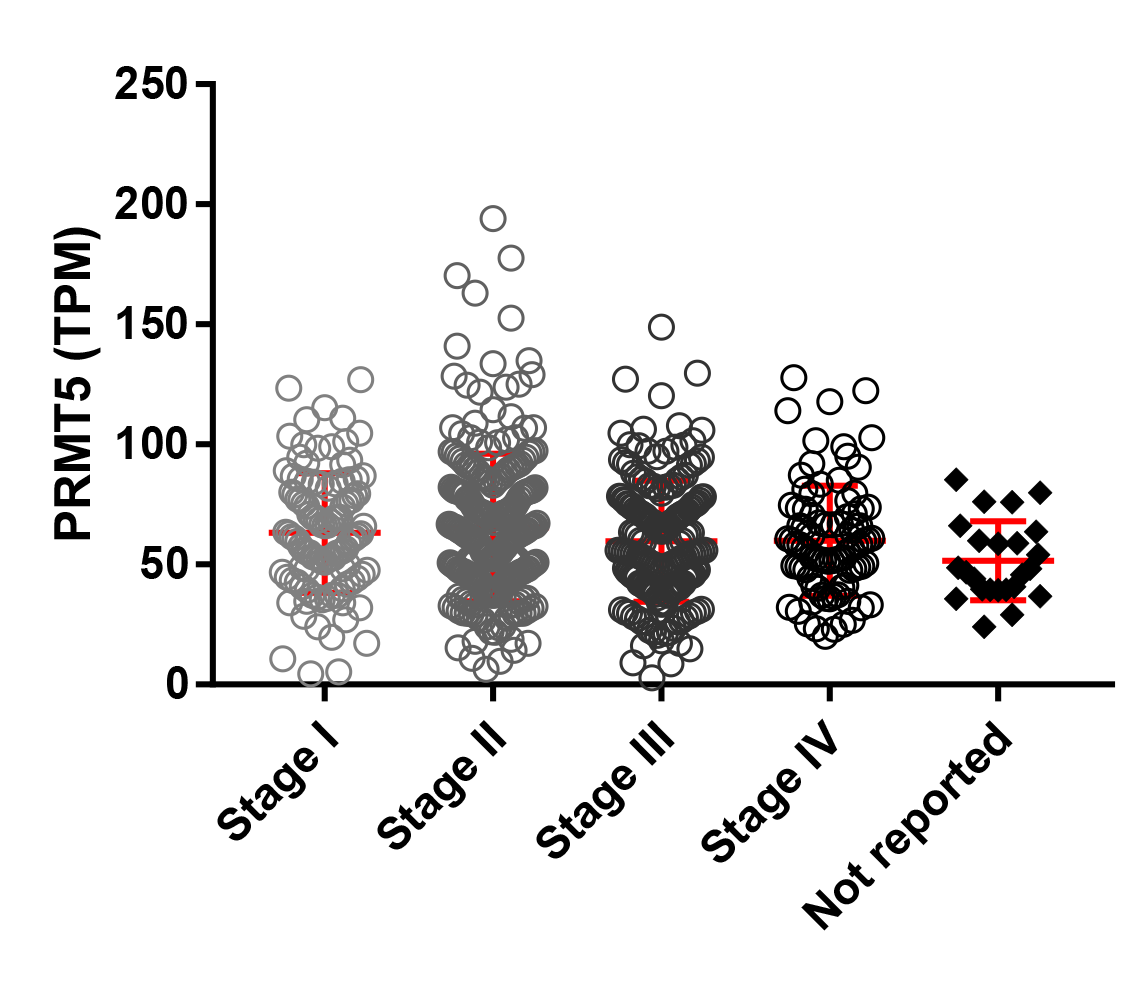

Supplement: Supplementary file 7 — Supplementary Figure S6 [file 41419_2019_1487_MOESM7_ESM.tif]
