# Supplementary material for: NAA40 contributes to colorectal cancer growth by controlling PRMT5 expression
Source: Cell Death Dis. 2019 Mar 11;10(3):236. doi: 10.1038/s41419-019-1487-3 (PMC6411749; doi:10.1038/s41419-019-1487-3)
Supplement: Supplementary file 6 — Supplementary Tables [file 41419_2019_1487_MOESM6_ESM.docx]

| **Supplementary Table 1: Short hairpin RNA oligos for plasmid construction** | | | | |
| --- | --- | --- | --- | --- |
| **Name** | | **Forward** | | **Reverse** |
| SCR | | CCGGTTCTCCGAACGTGTCA CGTTTCTCGAGAAA CGTGAC ACGTTCGGAGAATTTTTG | | AATTCAAAAATTCTCCGAACG TGTCACGTTTCTCGAGAAACG TGACACGTTCGGAGAA |
| NAA40-KD1 | | CCGGGGAAGTTCCTCATACA GATCCCTCGAGGGATCTGTA TGAGGAACTTCCTTTTTG | | AATTCAAAAAGGAAGTTCCTC ATACAGATCCCTCGAGGGAT CTGTATGAGGAACTTCC |
| NAA40-KD2 | | CCGGGAAGGTTATGTTAACA GTATTCTCGAGAATACTGTTA ACATAACCTTCTTTTTG | | AATTCAAAAAGAAGGTTATGT TAACAGTATTCTCGAGAATAC TGTTAACATAACCTTC |
| **Supplementary Table 2: Short interfering RNA oligos for transient transfection** | | | | |
| **Name** | **Forward** | | **Reverse** | |
| SCR | UUCUCCGAACGUGUCACGUTT | | ACGUGACACGUUCGGAGAATT | |
| NAA40-KD1 | CUUUCCCAGUGUUCAAGAATT | | UUCUUGAACACUGGGAAAGTT | |
| NAA40-KD2 | GAAGGUUAUGUUAACAGUATT | | UACUGUUAACAUAACCUUCTT | |

| **Supplementary Table 3: qRT-PCR and ChIP primer sequences** | | |
| --- | --- | --- |
| **Gene name** | **Forward** | **Reverse** |
| *NAA40* | TGGTGCCTACCAGTTCTTCA | CTCCGGCTCAGGATCTCATA |
| *β-actin* | GGCATCCTCACCCTGAAGTA | AGGTGTGGTGCCACATTTTC |
| *PRMT5* | TTGCCGGCTACTTTGAGACT | ACAGATGGTTTGGCCTTCAC |
| *PRMT7* | AGTGTGTGTACTTCCTGCCA | TAGTCATCGTGGTGGGCTAC |
| *EIF4E* | GGTGCCTGACATCGTGTTTT | AACACAGAGCCCCAACAGTA |
| *FGFR3* | CTGTACGTGCTGGTGGAGTA | GCAGGTGTCGAAGGAGTAGT |
| *CDKN1A* | TGTCTTGTACCCTTGTGCCT | CTGAGAGTCTCCAGGTCCAC |
| *RBL2* | TTGTTGGGTGCTTTTTATATATGC | TTTCCATAAACTAAGTCCAAAGCA |
| *CDH1* | TTGCACCGGTCGACAAAGGAC | TGGAGTCCCAGGCGTAGACCAA |
| *THOC1* | TGTGGACGGATTCAGCTCTT | GGGTGCTTTCCTGCTCATTT |
| *PRMT5* ChIP | ATAGCTGACACACTAGGGGC | CTAGTCTGCCCTTCTCCGTC |
