# Supplementary material for: NAA40 contributes to colorectal cancer growth by controlling PRMT5 expression
Source: Cell Death Dis. 2019 Mar 11;10(3):236. doi: 10.1038/s41419-019-1487-3 (PMC6411749; doi:10.1038/s41419-019-1487-3)
Supplement: Supplementary file 8 — Supplemental Figure legends [file 41419_2019_1487_MOESM8_ESM.docx]

**NAA40 contributes to colorectal cancer growth by controlling PRMT5 expression**

Christina Demetriadou^1^, Demetria Pavlou^1^, Fotios Mpekris^3^, Chara Achilleos^2^, Triantafyllos Stylianopoulos^3^, Apostolos Zaravinos^4^, Panagiotis Papageorgis^3,4^ and Antonis Kirmizis^1^

**Supplementary Figure S1: NAA40 expression is not correlated with tumor stage. (A)** Representative immunofluorescence images of NAA40 staining in CRC patient tissues corresponding to different adenocarcinoma stages (n=232). Green-FITC staining was used against NAA40 and blue DAPI staining was used to visualize cell nuclei. Scale bar, 50 μm. **(B)** Meta-analysis of *NAA40* expression levels extracted from the TCGA data portal in CRC adenocarcinoma specimens with different TNM stages (n= 647).

**Supplementary Figure S2: Loss of NAA40 protein levels in xenograft tumours*.*** Representative immunofluorescence NAA40 staining (green) images of tumors derived from SCR and NAA40-KD mouse xenografts after administration of dox (+dox) or PBS (-dox). DAPI staining (blue) was used to visualize cell nuclei. Scale bar, 200 μm.

**Supplementary Figure S3: NAA40 controls PRMT5 expression in SW480 colon cancer cells. (A)** Western blot analysis of protein extracts from the indicated SW480 engineered cell lines using antibodies against H4R3me1, H4R3me2a, H4R3me2s and total histone H4 as loading control. The densitometry numbers below each blot define the normalized levels of H4R3me1, H4R3me2a and H4R3me2s against H4 relative to SCR cells. **(B)** Western blot analysis of cell extracts derived from dox treated SCR or NAA40-KD SW480 cells using antibodies towards NAA40, PRMT5, PRMT7 and β-actin as loading control. The values below each blot were calculated by densitometry analysis of NAA40, PRMT5 and PRMT7 bands relative to SCR control after normalization with β-actin. **(C)** qRT-PCR analysis of mRNA levels of *NAA40*, *PRMT5* and *PRMT7* normalized to *β-actin* in the indicated SW480 cells. Error bars represent the mean ± s.d of three biological replicates. Unpaired two-tailed Student’s t-test was used (ns=no significance, **p<0.01, ***p<0.001). **(D)** qRT-PCR analysis of mRNA levels in SW480 cells for the indicated genes. The results were normalized to *β-actin* mRNA and represent the average from three independent experiments. Unpaired two-tailed Student’s t-test was used (*p<0.05, **p<0.01).

**Supplementary Figure S4: NAA40 regulates PRMT5 expression in SW620 colon cancer cells. (A)** Western blot analysis of protein extracts from the indicated SW620 stable cell lines using antibodies against H4R3me1, H4R3me2a, H4R3me2s and total histone H4 as loading control. The densitometry numbers below each blot define the normalized levels of H4R3me1, H4R3me2a and H4R3me2s against H4 relative to SCR cells. **(B)** Western blot analysis of cell extracts derived from dox treated SCR or NAA40-KD SW620 cells using antibodies towards NAA40, PRMT5, PRMT7 and β-actin as loading control. The values below each blot were calculated by densitometry analysis of NAA40, PRMT5 and PRMT7 bands relative to SCR control after normalization with β-actin. **(C)** qRT-PCR analysis of mRNA levels of *NAA40*, *PRMT5* and *PRMT7* normalized to *β-actin* in the indicated SW620 cells. Error bars represent the mean ± s.d of three biological replicates. Unpaired two-tailed Student’s t-test was used (ns=no significance, **p<0.01, ***p<0.001). **(D)** qRT-PCR analysis of mRNA levels in SW620 cells for the indicated genes. The results were normalized to *β-actin* mRNA and represent the average from three independent experiments. Unpaired two-tailed Student’s t-test was used (*p<0.05, **p<0.01).

**Supplementary Figure S5: NAA40 depletion reduces PRMT5 protein levels in xenograft tumors.** Representative immunofluorescence PRMT5 staining (green) images of SCR and NAA40-KD xenograft tumors derived from mice receiving dox (+dox) or PBS (-dox). DAPI staining (blue) was used to visualize cell nuclei. Scale bar, 200 μm

**Supplementary Figure S6: PRMT5 expression is not correlated with tumor stage.** Meta-analysis of *PRMT5* expression levels extracted from the TCGA data portal in CRC adenocarcinoma specimens with different TNM stages (n= 647).
